# Epigenetic odyssey to decrypt the hidden code for sustainable brassica production: enhancing yield, stress resilience and nutritional quality

**DOI:** 10.3389/fpls.2025.1696718

**Published:** 2026-02-02

**Authors:** R. Shubhra Maithreyi, Sugyan Preet, Kunal Tanwar, Puja Chakraborty, Isha Gupta, Rachana Verma, Jyotsna Bharti, Arulprakash Thangaraj, Rashmi Kaul, Tanushri Kaul

**Affiliations:** Nutritional Improvement of Crops Group, International Centre for Genetic Engineering and Biotechnology, New Delhi, India

**Keywords:** brassica crops, epibreeding, regulatory control, epigenetic editing, mitoepigenetics, stress resilience

## Abstract

High demand for agricultural products together with the changing climate imposes an urgency for improving crop productivity and quality. The Brassica family has evolved as a globally significant oilseed crop due to its multifaceted application for edible oils, seed meals, and biodiesel production. However, its wide-scale crop production is limited due to the advent of several external stressors. Development of resilient *Brassica* crops requires recognition of the chromatin state complexes to fine tune the transcriptional machinery. Epigenetic modification through DNA methylation, histone modification, RNA directed gene silencing, and chromatin remodeling plays a major role in modulating flowering time, gametogenesis, embryogenesis, seed development and whole genome duplication to shape key agronomical traits. In conjunction, recent progress in the field of sequencing technologies and genome editing have led to the characterization of key epigenetic markers for identifying crucial agronomic traits and subsequent molecular designing. Therefore, the current review details the mechanism and application of the recent epigenetic approaches that have contributed for the generation of climate-smart *Brassica* family.

## Introduction

*Brassica* genus is an important oilseed crop developed from interspecific hybridization and chromosome doubling of the diploid progenitors (*B. rapa*, *B. nigra*, and *B. oleracea*) to form allopolyploid species (*B. napus*, *B. juncea*, & *B. carinata*) (Kang et al., 2021). Growth and development of the *Brassica* genus is significantly hampered by the external stressors due to the changing climatic conditions. Development of superior lines with improved stress resilience and yield production requires in-depth understanding of the functional modulators for refining respective regulatory pathways. For instance, Canola epilines were selected for improved energy efficiency and drought tolerance by assessing the changes in the histone methylation patterns (Verkest et al., 2015). A possible strategy for developing adaptive cultivars may include characterizing epigenetic regulation along with molecular modification. Epigenetic variations accountable for improved yield, enhanced resistance, and cultivation of elite varieties have been reported in several crops including wheat, rice, soybean, and maize (Liu et al., 2017a; Deng et al., 2017; Raju et al., 2018; Begcy and Dresselhaus, 2018). *Brassica* genus underwent whole genome duplication to create allopolyploid species from their diploid progenitors (Triangle of U) which can be recreated experimentally and can be an ideal model system for epigenetic research. *Brassica* species are useful for epigenetic modifications due to their variation in ploidy level, genome size and the organization of heterochromatin (Braszewska-Zalewska et al., 2010). The epigenetic marking superimposed on the *Brassica* chromosomes has the ability to regulate gene expression, ontology, and environmental responses. These very marks affect the structure of chromatin, including cytosine methylation of DNA and histone modifications. *Brassica’s* complex genomic make-up, its agricultural relevance as an important oilseed crop, and dynamic metabolic processes such as flowering time makes it a unique system to study the impact of epigenetic modification on speciation, adaptation and subsequent crop improvement.

Determined by their accessibility, gene sequence is transcriptionally modulated through its chromatin organization, where densely packed heterochromatin regions are less accessible to transcription than the less compact euchromatin region (Bender, 2004). Epigenetic modification alters the probability of a gene expression through reversible transgenerational inheritance. These changes to the chromatin are facilitated through the action of small RNA (sRNA) dependent and independent DNA methylation, histone modification, chromatin modification, and RNA-dependent gene silencing (RdGS) (Meyer, 2015). Epigenetic markers create a lasting impact by controlling several developmental pathways in plants including growth, flowering time, embryogenesis and production of gametes, stress resilience, and other changes in morphology (Sena et al., 2024). An epigenetic modification alters the genetic make-up by influencing activation and repression; it does not directly change the DNA sequence, thereby restoring the original epigenetic state. In comparison to epigenetic marks erasure in early developmental stages of mammals, plants harbor the capability to pass these epigenetic markers through somatic cell division or germline (Meyer, 2015; Baulcombe and Dean, 2014).

Improvements in the high-throughput sequencing technology has evolved our understanding of plant regulatory networks and accelerated identification of important agronomic characters (Le Nguyen et al., 2019). Recent progress of these well-established methods like bisulfite sequencing (BS-Seq) for identification of 5-methylcytosine (5mC), methylated RNA immunoprecipitation (MeRIP) sequencing for mapping the location of N6-methyladenosine (m^6^A), as well as, chromatin immunoprecipitation sequencing (ChIP Seq) for characterizing histone modification has enabled recognition of distribution pattern of epigenetic modifications (Wang et al., 2021). Additionally, advances in the epigenetic editing research have enabled targeted genome modifications in the plant genome thereby dramatically transforming crop improvement (Lee et al., 2020). The current review outlines the epigenetic modifications and the related mechanisms responsible for regulating the plant’s fundamental mechanisms, along with the implementation of advanced epigenomic tools in generating climate-resilient *Brassica* varieties.

## DNA methylation

The process of DNA methylation is regulated by a set of genes that directly or indirectly influence the methylation status via chromatin remodeling (Amoah et al., 2012). DNA modification through covalent addition of a methyl (CH3) group to the cytosines (5-mC) occurs primarily in the CG, CHG (symmetric), and CHH (asymmetric) motifs (H=A, C, or T) present in the repetitive regions of the plant genome enriched with transposable elements and centromeric repeats (Pikaard and Scheid, 2014; Gallego‐Bartolomé, 2020). DNA methylation patterns are established either through *de novo* or maintenance methylation by modifying previously unmethylated sites or maintaining preceding methylation patterns imparted from the parent strand to the daughter strand (Pikaard and Scheid, 2014; Dinkar et al., 2024). Maintenance methylation is symmetrically distributed in CG and CHG motifs in the antiparallel strands of DNA using DNA methyltransferases - DNA Methyltransferase 1 (MET1) and Chromomethylase 3 (CMT3), respectively (Wambui Mbichi et al., 2020; Cao et al., 2023). Following replication, MET1 recognises hemi-methylated CG sites and methylates unmodified cytosine on the nascent daughter strand utilizing S-adenosyl-L-methionine (SAM) as the methyl donor (Zhang et al., 2018b). Variation in Methylation (VIM) proteins act as co-factors by mediating loading of MET1 to methylate the complementary CG target sites to form fully methylated strands (Shook and Richard 2014; Kim et al., 2014).

Alternatively, CHG methylation is primarily maintained by the enzyme Chromomethylase 3. CMT3’s are classified by the presence of Bromo-Adjacent Homology (BAH) and Chromatin Organization Modifier (CHROMO) domain. BAH and Chromo-domains recognize nucleosomes containing histone H3 lysine 9 dimethylation (H3K9me2) and facilitate CHG methylation (Du et al., 2012; Du et al., 2023). H3K9 methyltransferases, Suppressor of Variegation 3–9 Homologs (SUVH), namely, SUVH4/Kryptonite (KYP), SUVH5, and SUVH6 catalyzes binding to methylated CHG through their SET and Ring Associated (SRA) domains, leading to dimethylation (Liu et al., 2007). Therefore, CMT3 and SUVH4 enforce a self-regulatory feedback loop between H3K9me2 and CHG methylation to maintain the constitutive heterochromatin region (Bewick et al, 2017; Law and Jacobsen, 2010; Fang et al., 2022). In *Arabidopsis*, maintenance of pre-existing methylation is also conferred with the help of Domains Rearranged Methyltransferase (DRM) proteins, particularly, DRM1 and DRM2 (Cao and Jacobsen, 2002a). Cao and Jacobsen reported the functional redundancy of DRM and CMT3 loci for maintaining patterns of CHG methylation in *Arabidopsis* (Cao and Jacobsen, 2002b). In comparison, Brassicaceae species have reported lower levels of genomic and genic mCG, genome-wide mCHG, and gene body methylation (gbM) genes (Bewick et al., 2017).

**Figure 1:**
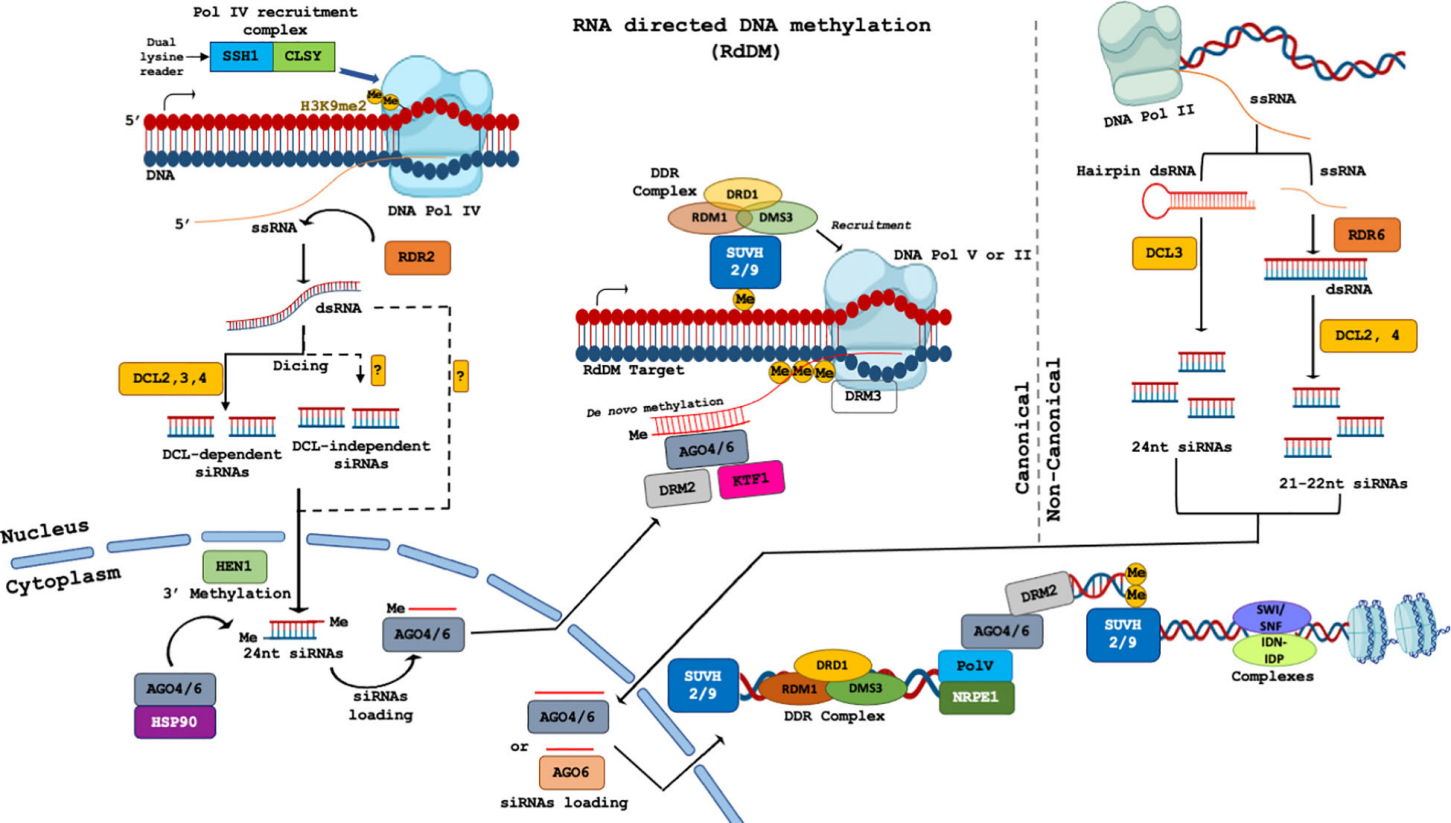
Canonical and non-canonical RNA directed DNA methylation (RdDM) in plants. SSH1 – Suppressor of gene silencing 1, CLSY – CLASSY chromatin-remodeling factor, RDR2 – RNA-Dependent RNA Polymerase 2, DCL – Dicer-Like (RNase III endonucleases), HEN1 – HUA ENHANCER 1 (small RNA methyltransferase), AGO – ARGONAUTE protein, HSP90 – Heat Shock Protein 90, DRD1 – DEFECTIVE IN RNA-DIRECTED DNA METHYLATION 1 (chromatin remodeler), RDM1 – RNA-DIRECTED DNA METHYLATION 1, DMS3 – DEFECTIVE IN MERISTEM SILENCING 3 (structural maintenance of chromosomes protein), SUVH2/9 – SU(VAR)3–9 HOMOLOG PROTEIN 2/9 (H3K9 methyltransferases), DRM3 – DOMAINS REARRANGED METHYLTRANSFERASE 3, KTF1 – KOW DOMAIN-CONTAINING TRANSCRIPTION FACTOR 1, Pol V – Plant-specific DNA dependent RNA Polymerase V, Pol IV – Plant-specific DNA dependent RNA Polymerase IV, Pol II – DNA dependent RNA Polymerase II, NRPE1 – Nuclear RNA Polymerase E1 (largest subunit of Pol V), SWI/SNF complex – SWItch/Sucrose Non-Fermentable chromatin-remodeling complex IDN/IDP complex – INVOLVED IN DE NOVO/INVOLVED IN DE NOVO PLASMID complex, ssRNA – single stranded RNA, dsRNA – double stranded RNA, Me – Methyl group (Samantara et al., 2021).

*De novo* methylation of the CHH motifs occurs through the chromomethylase 2 (CMT2) and RNA-directed DNA methylation (RdDM) pathways (**Figure 1**). CMT2 methylates CHH sites present in the long heterochromatic TEs, independent of the RdDM pathway, probably through interactions with H3K9me2 sites (Zemach et al., 2013). RdDM pathways involve the formation of small-interfering RNAs (siRNAs) and the recruitment of the DNA methylation complex through these siRNAs to methylate the target sites (Matzke and Mosher, 2014). In the canonical RdDM pathway, RNA Polymerase IV is recruited to the heterochromatic loci by the Sawadee Homeodomain Homolog 1 (SHH1) & CLASSY (CLSY) proteins for the synthesis of single-stranded RNAs (ssRNAs). Consequently, these 30-45nt long ssRNAs are converted to 26–45 nt double-stranded RNAs (dsRNAs) by RNA-dependent RNA polymerase 2 (RDR2) (Cuerda-Gil and Slotkin, 2016; Gallego‐Bartolomé, 2020). The dsRNAs are then cleaved by Dicer-Like 3 (DCL3) into 24-nt long siRNAs which are methylated by methyltransferase HEN1 (Hua Enhancer 1) in the cytoplasm for stabilization (Pikaard and Scheid, 2014). The single strand of the siRNA is loaded into Argonaute 4 (AGO4) or closely related AGO6 proteins to form siRNA-AGO complexes and relocated to the nucleus for RdDM effector complex assembly (Wambui Mbichi et al., 2020). In the second phase of the RdDM pathway, RNA Polymerase V transcribes a non-coding transcript (ssRNAs complementary sequence) which remains attached at the locus of origin and functions as the scaffold RNAs that interact with the siRNA-AGO complexes through sequence complementarity. Pol V transcription is dependent on the DDR Complex - Defective in RNA-directed DNA Methylation 1 (DRD1), Defective in Meristem Silencing 3 (DMS3), and RNA-directed DNA Methylation 1 (RDM1) for its association with the chromatin. Ensuing, the AGO4-bound siRNA binds to the Pol V transcript and recruits Domains Rearranged Methyltransferase 2 (DRM2) for DNA methylation of the homologous RdDM loci. DNA methyl-readers, SUVH2 and SUVH9, function as adaptors to regulate Pol V occupancy to the chromatin (Matzke and Mosher, 2014; Liu et al., 2014a).

Besides the Pol IV-dependent 24-nt siRNA directed RdDM pathway, non-canonical pathway involves biogenesis of sRNAs from different sources such as Pol-II-derived mRNAs to direct the canonical RdDM pathway. Cleavage of the dsRNAs by various Dicer-like (DCL) proteins yields the sRNAs which trigger post-transcriptional gene silencing of the target loci through mRNA cleavage or translational repression. The different forms for non-canonical RdDM reported in the plant systems are (a) Inverted repeats and microRNA (miRNA) directed pathways which involve Pol II-DCL3 RdDM, (b) RDR6 RdDM pathway, (c) RdR6-DCL3 RdDM pathway, (d) Pol IV-NERD (Needed for RDR2 independent DNA methylation) RdDM pathway, (e) double strand break repairs through RdDM proteins, and (f) Dicer-independent RdDM pathway, described in detail by Cuerda-Gil and Slotkin (2016). Loading of these sRNAs into AGO4 initiates DRM2 dependent methylation of the homologous gene loci and causes transcriptional gene silencing (TGS) (Gallego‐Bartolomé, 2020).

The balance and pattern of 5-mC is maintained by both DNA methylation and demethylation. Demethylation functions to remove promiscuous methyl groups to confer the correct methylation pattern and activate/reset specific genes (Zhu et al., 2025). Active DNA demethylation involves a subfamily of DNA glycosylase which removes the CH3 group through base excision repair pathway (Zhu, 2009). When an endogenous gene promoter codes for siRNAs, it triggers gene silencing throughout RdDM dependent methylation. To prevent silencing of the endogenous gene, Repressor of Silencing 1 (ROS1) gene encodes for a DNA glycosylase (Ponferrada-Marín et al., 2009). In *Arabidopsis*, ROS1 initiates 5-mC excision by cleaving the phosphodiester bond at the removal site and leaving behind a gap which is processed as part of the base excision repair pathway by DNA polymerase and DNA ligase I (Ponferrada-Marín et al., 2009). Demeter-like proteins - DML2 and DML3, along with ROS1 and Demeter (DME) proteins contain DNA glycosylase domain to prevent DNA hypermethylation (Liu and Lang, 2020).

Advent of transcriptome-wide sequencing and mapping strategies paved the way for characterization of chemical modifiers of cellular RNA in plants. These mRNA modifications, collectively referred to as epitranscriptomes, modulates RNA metabolism and influences gene regulation. Epitranscriptomes refers to the biochemical changes including *N*^6^-methyladenosine (m^6^A), N^1^ methyladenosine (m^1^A), *N*^4^-acetylcytidine (Ac4C), 5-methylcytosine (m^5^C), 5-hydroxymethylcytosine (hm^5^C), m^7^G, and pseudouridine (ψ) modifications of the cellular RNAs (Shen et al., 2023). Among these, N^6^-methyladenosine (m^6^A) is the most abundant RNA modification influencing the polyploid genome architecture and stress tolerance patterns across the *Brassica* family.

## Histone modifications

### Histone acetylation

Histone acetylation involves covalent addition of acetyl group to lysine residues, catalyzed by histone acetyltransferase (HAT). Since acetyl group has negative charge addition of this group nullifies the electrostatic force of attraction between positively charged histone and the negatively charged DNA resulting in relaxation of the supercoiled DNA and transcription activation (Suto et al., 2000). Specific lysine residues present at N-terminal of histones are susceptible to acetylation such as in *Arabidopsis*, lysine residues of histone H3(K9, K14, K18, K23, and K27) and H4 (K5, K8, K12, K16, and K20) undergo acetylation (Earley et al., 2007). *Arabidopsis* has 12 histone acetyltransferases grouped in four families: the GNAT/HAG (GCN5-related N-acetyltransferase), the MYST (Moz, Ybf2/Sas3, Sas2, and Tip60)/HAM, the p300/CBP/HAC (histone acetyl transferase) and the TAFII250/HAF families (Fina et al., 2017). The orthologues of *Arabidopsis* GCN5 cause acetylation predominantly at H3K9 and H3K14 (Xue et al., 2024). The CBP/p300 family HISTONE ACETYLTRANSFERASE 1 (HAC1) promotes histone acetylation to induce flowering via a CONSTANS-dependent activation of FT (FLOWERING LOCUS-T) and SOC1 (SUPPRESSOR OF OVEREXPRESSION OF CONSTANS 1) and also functions as HAC1/5 MEDIATOR complex to regulate flowering time (Liang et al., 2025; Guo et al., 2021). HAF2 (Histone Acetyltransferase of the TAFII250 Family 2) gene regulates circadian rhythm through H3 acetylation at PRR5 (Pseudo-Response Regulator 5) and LUX (LUX ARRHYTHMO) loci (Lee and Seo, 2018). It also controls H3K14ac and H3K23ac in ethylene response (Chen et al., 2025).

### Histone deacetylation

Histone deacetylation is the reversible removal of acetyl group from the lysine residues of the core histones by histone deacetylase (HDAC) and is responsible for gene transcriptional repression. In *Arabidopsis* and *Brassica rapa*, 18 and 20 HDACs have been identified respectively and categorized into three main families; HDT/HD2 family (histone deacetylase 2), RPD3/HDA1 family (Reduced Potassium Deficiency 3/histone deacetylase 1), and Sir2 family (Silent information Regulator2) (Hollender and Liu, 2008; Hu et al., 2009; Eom and Hyun, 2021). In *Arabidopsis*, HD2A, HD2B, and HD2C members of the HD2 family, play a significant role in plant developmental pathways and are predominantly expressed in leaves, embryonic tissues, flower and siliques whereas the HD2D is expressed mainly in stem and floral parts (Wu et al., 2000, Wu et al., 2003; Zhou et al., 2004; Tahir et al., 2022). HD2C deacetylates H3 histone at K9 and K14 residues and also interacts with HDA6 to regulate gene expression in roots (Luo et al., 2012). HDA5 (HISTONE DEACETYLASE5) one of the members of RPD3/HDA1, plays an important role in early flowering by deacetylating H3 histone of FLC chromatin and also works in association with HDA6 in multiple developmental pathways (Luo et al., 2015). Deacetylases of the Sir2 family mediates NAD^+^ dependent deacetylation of different substrates including histones (Tang et al., 2022).

### Histone methylation

Histone methylation is the process of addition of methyl functional groups to the arginine and lysine side chains by histone lysine methyltransferase (HKMT). In *A. thaliana*, H3K4me3 and H3K36me3 associated with highly expressed genes, but H3K27me3 associated with lowly expressed genes or tissue-specific genes (Turck et al., 2007; Zhang et al., 2007, Zhang et al., 2009; Roudier et al., 2011). In *Brassica rapa*, chromatin regions marked with either H3K34me3 or H3K36me3 only shows low tissue-specific gene expression but when both are present in the regions show higher levels of expression of tissue-specific genes (Mehraj et al., 2021a). Chromatin accessibility is maintained by histone methyltransferases (HKMTases) consisting of the SET domain and histone demethylases containing the jmjC and LSD domains. Lysine-specific demethylase 1 (LSD1) involved in reversing mono- and di-methylated lysine substrates using FAD as co-factor, on the other hand Jumonji C domain-containing (JMJD), a lysine demethylases remove trimethyl mark from lysine residues at H3K9 and H3K36.

### Histone ubiquitination

Histone ubiquitination process involves covalent addition of ubiquitin group composed of a 76-amino acid polypeptide, to lysine residue of histone protein via sequential action of three enzymes, E1- activating, E2-conjugating and E3-ligating enzymes (Saracco et al., 2009). The enzyme complex mediates both types of ubiquitylation (mono- or poly-) and substrate specificity. H2 mono-ubiquitylation have been well studied i.e., H2AK119ub1 participates in gene silencing via Polycomb pathway, and H2BK123ub1 participates in transcriptional initiation and elongation (Wang et al., 2004a). Besides H2Aub1 and H2Bub1, ubiquitinylation of H1, H3, and H4 are also observed in *Arabidopsis* (Manzano et al., 2008; Saracco et al., 2009). The modification is removed via the action of a de-ubiquitinizing enzyme such as OTU1 (Ovarian tumor domain-containing deubiquitinating enzyme 1) which removes monoubiquitin mark from H2B to inhibit DA1 and DA2 genes to regulate seed and organ size (Keren et al., 2020). Deubiquitination and histone acetylation also works in a coordinate manner because UBP5 (Ubiquitin Specific Protease 5) and histone acetyltransferase form an integral part of PEAT (PWWP, EPCR, ARID, TRB subunits) complex for an active chromatin formation in *Arabidopsis thaliana* (Zheng et al., 2023). This shows that ubiquitination may work in coordination with other histone modifications to regulate gene expression and developmental pathways.

### Histone sumoylation

SUMO (small ubiquitin-related modifier) is a ubiquitin-like protein having 18% similarity to ubiquitin primary sequence (Melchior, 2000). It is added to lysine residue of histone via sequential action of E1, E2 and E3 enzymes similar to ubiquitylation and denotes repressive functions (Bannister and Kouzarides, 2011). In *Arabidopsis*, sumoylation is mediated by heterodimeric E1- consisting of single SUMO-ACTIVATING ENZYME (SAE)-2 and either of two SAE1 isoforms (SAE1a and SAE1b), transferred to single E2- SUMO-CONJUGATING ENZYME (SCE)-1 (Kurepa et al., 2003; Saracco et al., 2007). This complex is conjugated with the help of three E3 type ligases- SAP and MIZ1(SUMO E3 ligase, SIZ1; SAP AND MIZ1 DOMAIN-CONTAINING LIGASE1), METHYLMETHANESULFONATE-SENSITIVE (MMS)-21 or HIGH PLOIDY (HPY)-2, and PROTEIN INHIBITORS of ACTIVATED STAT-LIKE (PIAL)-1 and PIAL-2 (Miura et al., 2005; Cheong et al., 2009; Huang et al., 2009; Ishida et al., 2009; Tomanov et al., 2014). Based on proteomic studies in *Arabidopsis thaliana*, histones such as H2A variants (HTA6,7) and H2B variants (HTB2,4,9) may act as the targets for sumoylation (Augustine and Vierstra, 2018), but proper validation has not been done in any of the Brassicaceae family members.

### Histone phosphorylation

Histone phosphorylation involves addition of phosphate group to serine, threonine and tyrosine of N-terminal histone tails as well as linker histone H1 by histone kinases (Rossetto et al., 2012). The action of kinases is reversible due to histone phosphatases which removes the phosphate group. In *Arabidopsis*, three Aurora homologs are found (AtAUR1, AtAUR2 and AtAUR3) and all of them phosphorylates H3S10 *in vitro*, but during mitosis, only AtAUR3 showed a localization dynamic similar to H3S10ph (Demidov et al., 2005; Kawabe et al., 2005). In *Arabidopsis thaliana*, immunolocalization studies show that H3S10ph modification participates during cell division in root meristem (Kelemen et al., 2025). Other than AUR family, nuclear localized PYRUVATE KINASE (PK)-6,7 and 8 modulate H3T11ph for transcriptional activation of FLC (Flowering Locus C), PRE1(Paclobutrazol Resistance 1), MYB73 (MYB Domain Protein 73), TCP4 (Teosinte Branched1/Cycloidea/PCF4) and TCP10. and consequently, regulate plant growth. H3T11ph accumulation is dependent on glucose availability which directly affects PK6 levels required for FLC dependent pathway for flowering (Hu et al., 2024). Phosphorylation of H2A at serine 95 mediated by MLK (mixed lineage kinase) 3 and 4, is essential to induce flowering and H2A.Z deposition in *Arabidopsis* (Huang et al., 2021), but MLK4 also phosphorylates H3T3 during flowering by silencing the FLC/MAF (FLOWERING LOCUS C/MADS AFFECTING FLOWERING) at transcriptional level (Wang et al., 2021).

## Chromatin remodeling complexes

The basic characteristics of chromatin remodellers for example, their preference to bind to nucleosomes rather than DNA, and the presence of a single catalytic ATPase remain the same in plants, despite modulating multiple cellular processes (Clapier et al., 2017; Chen et al., 2017). The complexes mediate nucleosome positioning, ejection or exchange of histones via the energy obtained from ATP hydrolysis (Clapier et al., 2017), thereby controlling both the packaging and unpackaging of DNA (Gioacchini and Peterson, 2017). Dissociation of nucleosomes can be achieved by chromatin remodellers; i.e ATPases for instance, the SWI/SNF (switch/sucrose non-fermentable) complex, first characterized in yeast (Jerzmanowski, 2007; Becker and Workman, 2013). There are four families of chromatin remodeling complexes, based on the ATPase subunit composition: inositol requiring 80 (INO80), SWI/SNF, imitation switch (ISWI), and chromodomain helicase DNA-binding (CHD) complexes (Narlikar et al., 2013; Bartholomew, 2014, Yang et al., 2022). The INO80 subfamily in plants is the most recent addition to the SWI/SNF family of chromatin modifying factors, and its ATPase orthologues and homologues have been identified in yeast, flies and mammals (Jian et al., 2021; Morrison and Shen, 2009). SWI/SNF complex is evolutionarily conserved and plays key roles in defence against abiotic and biotic stresses. ISWI is an evolutionarily conserved chromatin remodeling protein subfamily, which exists in *S. cerevisiae*, multi-cellular organisms including fruit flies, plants and mammals. ISWI is involved in overall development and heat stress responses in *Arabidopsis*. CHD complexes are divided into three groups, while the group I CHD proteins participate in nucleosome positioning, the role of group II members is not known (Lu et al., 2020).

The SHPRH (sucrose non-fermenting 2 or SNF2) subfamily, present in mammals, responsible for epigenetic modifications, is characterized by the presence of conserved ATPase and RING (really interesting new gene) domains. *BrCHR39* belongs to the SHPRH family. The chromatin remodeling factor, *BrCHR39*/*Bra014815* homolog of *AtCHR39*, is known to positively regulate apical dominance by auxin signaling in *Brassica rapa* (Liu et al., 2025).

## RNA-directed gene silencing

RNA-directed gene silencing (RdGS) particularly RNA interference (RNAi) is a vital epigenetic mechanism which regulates gene expression at both transcriptional as well as post-transcriptional levels through small RNAs (sRNAs), majorly small interfering RNAs (siRNAs) and microRNAs (miRNAs) in plants (Wassenegger et al., 1994; Kelly and Aramayo, 2007; Borges and Martienssen, 2015). Sequence-specific silencing of the target genes is mediated by sRNAs through DNA methylation, histone modification or mRNA cleavage (Axtell, 2013). Mechanistically DICER-like (DCL) enzymes convert double-stranded RNA (dsRNA) into short RNAs of around 21–24 nucleotides in the first step of the RdGS pathway. The RNA-induced silencing complex (RISC) is subsequently formed by loading them onto AGO proteins (El-Sappah et al., 2021; Chen et al., 2025a). Gene silencing can occur transcriptionally through the RdDM pathway by silencing transcription and altering the state of the chromatin as well as post-transcriptionally through translational repression or mRNA cleavage, depending on the type of small RNAs. Therefore, gene silencing has been categorized into two primary modes: Transcriptional Gene Silencing (TGS) and Post-Transcriptional Gene Silencing (PTGS). TGS is mediated by DNA methylation at promoter or 5’UTR regions, leading to transcriptional repression. Alternatively, PTGS is operated through methylation within coding regions or via the RISC-mediated degradation of the target mRNA post-transcriptionally. Pri-miRNAs (Primary-miRNAs) which are transcribed by Pol II are processed by DCL1 into 21–22 nt miRNAs that will load into AGO1-containing RISC for translational inhibition or cleavage of target mRNAs (El-Sappah et al., 2021; Islam et al, 2022). Moreover, PTGS is guided by Natural antisense siRNAs (nat-siRNAs) and Trans-acting small interfering RNA (ta-siRNAs) as denoted in *Arabidopsis* and rice (Mirlohi and He, 2016; De Alba et al, 2013; Eamens et al., 2008; Chen et al, 2025a). RdDM promotes stable, heritable silencing of specified loci by guiding DNA methylation via siRNAs, connecting PTGS to TGS. These processes support genomic integrity, particularly by silencing transposable elements (TEs), reflecting the larger function of RdDM in defense, stress, and developmental changes.

RdGS the foundational plant epigenetic mechanism modulates gene regulation, to mitigate stress response, enhancing yield and nutrient acquisition in *Arabidopsis*, rice, and maize (Vermeersch et al, 2013; Brosnan et al, 2007; Mlotshwa et al., 2008). In *Arabidopsis*, microRNAs (miRNAs) played a major role in mollifying heat and oxidative stress. Downregulation of miR398 in response to oxidative stress permits up-regulation of its targeted gene CSD2 (copper/zinc superoxide dismutase). During heat stress, the upregulation of miR398 leads to the suppression of CSD genes resulting in the accumulation of ROS that is required for initiating the heat shock responses (Guan et al., 2013). Therefore, this underscores the intricacy of small RNA responses to environmental stimuli by this dynamic regulation. Although in *Brassica napus* the orthologous miRNAs are present but their regulatory function in respect to stress is less explored and needs future exploration. Another stress i.e. phosphate starvation is one of the key elements limiting the plant growth and development and miRNA family regulates how plants will react to the nutritional stress by suppressing the target gene expression at the post-transcriptional or translational stage. Moreover, miRNA assists the transportation of phosphate in plants by improving susceptibility to low phosphate (Pi) conditions. Under Pi deficiency miR399 is systemically translocated from shoots to roots and becomes transcriptionally activated, where it targets phosphate acquisition and systemic signaling by PHO2 (Phosphate Overaccumulator 2)-a ubiquitin-conjugating enzyme (Fujii et al., 2005; Chiou et al, 2006). *Arabidopsis* consists of six members of miR399 family i.e., miR399 a-f that are produced by inducing the plant roots, which are then swiftly transmitted to the shoots after Pi absorption (Pant et al, 2009, Pegler et al., 2021; Chen et al, 2025a). In rice, each of the seven miR399 family members have a distinct biological role, as shown by the inductive expression of Osa-miR399d, f, and j while other members were induced to accumulate in the shoots under Pi-deficient scenarios (Hu et al, 2015; Du et al, 2023; Chen et al, 2025a). However, it is still unknown how miR399 family genes (miR399a-c) affects rapeseed *Brassica napus* sensitivity under low Pi stress. Multifunctional roles of several miRNA to confer abiotic and biotic stress in *B. napus* has been reviewed by Li et al (Li et al., 2022a). Long non-coding RNAs are crucial for seed oil development as they modulate the activity of diacylglycerol transferase (DGAT) for lipid metabolism in high-oleic-acid rapeseed (Wang et al., 2023; Li et al., 2023a).

## Mitoepigenetics/MtDNA methylation

In plants, research on epigenetic modifications has traditionally focused on the nuclear genome. However, an emerging field termed “mitoepigenetics” investigates epigenetic modifications for regulation within the mitochondrial genome (Cao et al., 2021). Among the above-mentioned epigenetic modifications, mitochondrial DNA (mtDNA) has been reported to show evidence of cytosine methylation. Moreover, unlike the nuclear genome, plant mtDNA methylation has so far been described mainly in the CHH context (Muniandy et al., 2020). Epigenetic modulation of mtgenome has drawn interest because of its potential implications for mitochondrial genome stability, mtDNA replication, mitochondrial gene expression, and metabolic adaptation. Evidence from *Brassica* provides insight into how nuclear DNA methylation contributes to plant responses and developmental reprogramming (Tirnaz et al., 2022; Zhang et al., 2025; Hu et al., 2023). For instance, promoter demethylation of *BramMDH1* (mitochondrial malate dehydrogenase) enhanced its expression, contributing to cross-adaptation by improving heat tolerance and growth (Liu et al., 2017b). However, in mammals, alterations in mitochondrial DNA (mtDNA) methylation, particularly in regulatory elements such as the D-loop, have been associated with diverse physiological dysfunctions (Stoccoro and Coppedè, 2021). In cancer, these modifications can suppress transcription of respiratory chain genes (Dong et al., 2020); in metabolic disorders like type 2 diabetes, they reduce ATP production; and in neurodegenerative diseases, they compromise mitochondrial performance (Wagner et al., 2022). Environmental factors including hypoxia, high-fat diets, and exposure to pollutants have also been shown to reshape mtDNA methylation landscapes. If analogous processes exist in plants, mtDNA methylation may represent an underexplored regulatory layer influencing energy metabolism, growth, and stress adaptation. Considering its biological significance, dedicated research in this field has revealed limited evidence from plants which also points in mtDNA methylation direction. For instance, in rice, mtDNA shows distinct cytosine methylation patterns between leaves and grains, which align with their different energy requirements (Muniandy et al., 2020). Likewise, in *Arabidopsis* and maize, stress-induced changes in mitochondrial gene activity, along with indications of mtDNA cytosine methylation, highlight the possibility that this regulatory layer influences energy metabolism, growth, and responses to environmental stress (Bhanu et al., 2020).

Despite its potential importance, both the existence and functional relevance of mtDNA methylation in plants remain uncertain. A major source of this controversy is the presence of nuclear mitochondrial insertions (NUMTs), nuclear DNA fragments derived from ancient mitochondrial sequences (Zhong et al., 2025). For instance, in *Arabidopsis thaliana*, Chromosome 2 carries a large NUMT region nearly twice the size of the mitochondrial genome itself, sharing 99.93% sequence identity. Unlike mtDNA, which has been suggested to show CHH-specific methylation, NUMTs exhibit heavy methylation across all cytosine contexts. This overlap introduces technical artifacts, making it difficult to clearly distinguish genuine mtDNA methylation in sequencing-based studies. Recent work by Zhong et al. (2025) strengthens this argument, showing that mtDNA exhibits negligible methylation across all cytosine contexts, whereas the corresponding NUMT regions are heavily methylated. Their conclusions, based on methyl-CpG-binding domain (MBD) protein–based affinity enrichment coupled with next-generation sequencing, suggest that many earlier reports of extensive mtDNA methylation in plants may, in fact, reflect NUMT contamination rather than true mitochondrial modifications. Taken together, these uncertainties underscore a critical gap in our understanding. While mtDNA methylation could hold significant biological importance, the evidence so far remains inconclusive. This highlights the need for rigorous, reproducible studies using refined methodologies to establish whether mitoepigenetic regulation truly exists in plants and, if so, what role it plays in cellular adaptation and metabolism.

## Regulatory role of epigenetic modifications in plant development

### Flower development

The developmental shift from vegetative to flowering is controlled by a complex of regulatory pathways, including, vernalization (cool temperature), gibberellin-dependent (GA), photoperiodism (day length), thermosensing, and autonomous pathways (Wang et al., 2025a; Li et al., 2025). The endogenous and environmental signals modulate expression of FLOWERING LOCUS *C* (FLC) and its upstream activator FRIGIDA (FRI) to synergistically regulate floral transitions (Tadege et al., 2001; Choi et al., 2011; Ding and Chen, 2018; Calderwood et al., 2021). Before cold exposure, FLC work together with SHORT VEGETATIVE PHASE (SVP) and repress the gene expression of flowering promoter genes - FLOWERING LOCUS T (FT), FLOWERING LOCUS D (FD), and SUPPRESSOR OF OVEREXPRESSION OF CONSTANS 1 (SOC1)/AGAMOUS-LIKE 20 (AGL20). SIRTUIN 1 (SRT1) is known for deacetylation of H3K9ac by binding to the TSS sites of FT & SOC1 to delay flowering. In *Brassica rapa*, BraSRT1, 2 have been identified through phylogenetic and expression studies, but their functional role in repressing floral transition has not been explored yet (Wang et al., 2024; Liu et al., 2017c; Zhang et al., 2018b; Eom and Hyun, 2021). High-temperature induced FT repression denoted no correlation with presence of H2A.Z in *B. napus*, while the opposite held true in B. rapa. Alternatively, positive correlation between *Bn*FTA2 and H3K4me3 was observed at high temperature (Abelenda et al., 2023; Del Olmo et al., 2019).

**Figure 2:**
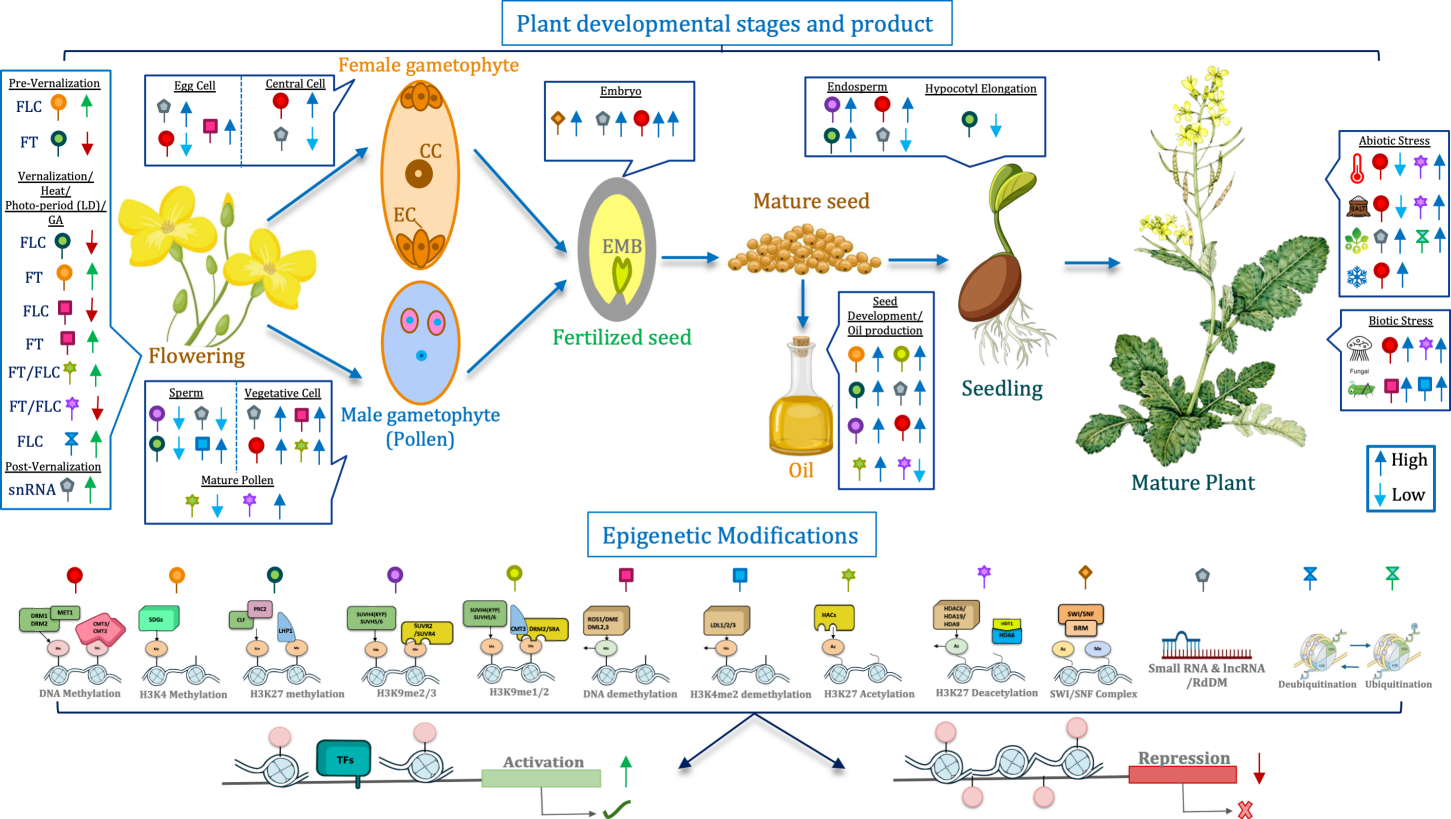
Schematic representation illustrating how key epigenetic modifications regulate development and stress perception in *Brassica* species through chromatin remodeling. These coordinated epigenetic switches ensure optimal plant growth, adaptive responses, and improved yield. Dark blue arrows indicate stages or processes where a particular epigenetic mechanism (such as DNA methylation, histone acetylation, or histone methylation) is highly active, whereas light blue arrows denote reduced or downregulated activity of the same mechanism. Green arrows mark the activation of target gene expression in response to favorable epigenetic states, while red arrows represent repression of gene expression mediated by repressive chromatin marks. Together, these interactions highlight how dynamic epigenetic regulation fine-tunes developmental transitions and enhances stress resilience in *Brassica*.

FRI represses floral transition through two mechanisms, one of them involves recruitment of the FRI-C complex which includes SWR1 chromatin remodeling complex and H3K3 methyltransferase Set Domain Group 8 (SDG8) to the FLC locus (**Figure 2**). This enhances FLC gene expression through active histone modifications such as H3K4me3, H3K36me3, and H3/H4 acetylation. Loss of function of the *Br*SDG8 represses *Br*FLCs expression and causes early bolting (Fu et al., 2020). Importance of SDG8 is also conserved in *B. napus* where knockdown/knockout of BnSDG8.A/C may cause early flowering (Jiang et al., 2018). Numerous studies have reported the role of histone modifications leading to epigenetic silencing of the FLC locus (He, 2009). Decreased FLC expression is mediated by long non-coding RNAs, COOLAIR (BrFLC2as), which accumulate during cold conditions (Li et al., 2024). Vernalization suppresses FLC expression to promote flowering through an epigenetic switch where active histone marks are replaced with H3K9, H3K27, and H4R3 methylation, H3K4 demethylation, and histone deacetylation in the FLC chromatin (Ahmad et al., 2010). *VERNALIZATION INSENSITIVE 3* (VIN3) forms a plant homeodomain-Polycomb repressive complex 2 (PHD-PRC2) to mediate histone H3K27 tri-methylation at the FLC locus (He and Amasino, 2005). UBIQUITIN-SPECIFIC PROTEASE 26 (UBP26), a deubiquitinase, enhances the levels of FLC to mediate late flowering by targeting H2B deubiquitination essential for accumulation of H3K36 trimethylation for transcriptional activation (Schmitz et al., 2009).

In *Brassica rapa*, H3K27me3 of BrFLC region regulates flowering during cold stress (Akter et al., 2019). Genome-wide analysis and transcriptomics study of H3K27me3 mark in *Brassica rapa* revealed that it regulates AGAMOUS-like loci during flower development (Payá-Milans et al., 2019). Curly Leaf (CLF), a PRC2 component, maintains FLC silencing by modulating H3K27me3 of the FT, SOC1, and SEP3 (SEPALLATA3) genes. Loss of function of BrCLF, led to increased BrFLC expression and early flowering in the mutants compared to the wild-type *B. rapa* (Poza-Viejo et al., 2024). During embryogenesis, epigenetic memory resets FLC expression through ELF6, a H3K27me3 demethylase, by removing epigenetic modifications in the next generation. Loss of BrELF6 conjured persistent H3K27me3 levels at the FLC locus causing early flowering in *Brassica* (Poza-Viejo et al., 2022).

Similar to its function in *Arabidopsis*, CONSTANS (BrCO) ortholog is regulated by the circadian clock in *B. rapa* and *B. napus* (Li et al., 2024; Kim et al., 2019; Chen et al., 2021). Cold induction causes enhanced expression of BrCKA2 and BrCKB4 (casein kinase II α and β subunits) through DNA demethylation by shortening the period of circadian clock associated 1 (BrCCA1) thereby increasing CO activity (Duan et al., 2017). HISTONE ACETYLTRANSFERASE 1 (HAC1) induces flowering via a CONSTANS-dependent activation of FT and SOC1 and also functions as HAC1/5 MEDIATOR complex to regulate flowering time (Guo et al., 2021; Liang et al., 2025). In *Brassica rapa*, BrHAC1 gene-silencing delayed bolting and flowering, likely to regulate FLC indirectly through upstream regulatory factors rather than direct FLC chromatin modification (Si et al., 2021). Additionally, GA boosts flowering through FT activation under long-day conditions and may also induce floral transition under short-day through SOC1 and LFY (Quiroz et al., 2021; Yu et al., 2012). In *Brassica rapa*, Jumonji H3K27me3 demethylase BrREF6 regulates flowering by modulating GA metabolic genes. Unlike *Arabidopsis* REF6, it does not alter the BrFLC gene expression. Evolutionary and expression studies have identified 60 SET {Su(var)3–9, enhancer of zeste and trithorax} domain containing proteins, 53 jumonji C (jmjC) domain-containing and 4 LSD in *B. rapa* but their functional validation has not been explored yet (Liu et al., 2019).

### Gametogenesis

In angiosperms, gametogenesis includes lineage-specific chromatin remodeling and DNA methylation. The female gametophyte comprises two gametes - the egg cell and the central cell. CG and CHG methylation remain stable in the female gametophyte, CHH methylation in the egg cell is modulated through DRM2 and RNA Polymerase V via the RdDM pathway in *Arabidopsis*. CHH pattern in the egg cell is hypothesized to play a key role in transcriptional control of the early embryo development (possibly due to *de novo* methylation by DRM2) (Chakraborty et al., 2021; Markulin et al., 2021). “Siren” loci produce highly abundant RdDM-associated siRNAs primarily in *B. rapa* ovules, possibly targeting PMEs, TMK receptor-like kinases, and DUF239 genes through trans-methylation (Burgess et al., 2022; Grover et al., 2018). Production of siRNA is dependent on Pol IV, RDR2, and CLASSY 3 (putative chromatin remodeler) and siren loci which is methylated in the ovules. Contrastingly, p4-dependent siRNA production was reduced in *B. rapa* denoting siRNA production through other independent pathways (Huang et al., 2013).

DNA methylation levels in meiocytes, microspores, and pollen of *B. rapa* revealed distinct patterns for CG and CHG methylation while the CHH methylation and sexual-lineage specific methylation loci remained similar to *Arabidopsis*. Combination of the methylome and transcriptome data denoted activation of long terminal repeat (LTR) TEs due to low CG & CHG methylation over TE bodies and dynamic CHH methylation at the flanking regions. Activation of MET1, CMT2, and CMT3 in the meiocytes offers possible conclusion of TE expression regulation through the RdDM mediated *de novo* methylation (Zhang et al., 2025). In *Arabidopsis*, Sperm nuclei display enhanced CG and CHG methylation in comparison to CHH methylation. In contrast, Vegetative Nuclei (VN) denotes opposing trends for DNA methylation (Mahmood et al., 2024). While CHH methylation mechanisms are conserved across Brassicaceae, CG and CHG methylation patterns differ in *B. rapa* in comparison to *Arabidopsis* (Zhang et al., 2025). Chromatin uncoiling promotes DME mediated demethylation and facilitates transcriptional activation of certain transposable elements (TEs) which promotes synthesis of epigenetically activated small interfering RNAs (easiRNAs) in *Arabidopsis*. These siRNAs migrate to the sperm cell from the VN and may epigenetically regulate zygote and endosperm following fertilization. Specific methylation loci in the male and female sex cells denoted weak correlation in *B. rapa* (Zhang et al., 2025).

Sperm cells undergo chromatin remodeling due to the genome-wide loss of H3K27me3 as a consequence of (a) active demethylation by Jumonji C (JmjC) family of H3K27 demethylases, (b) reduced PRC2 activity and ability to transcribe H3K27me3, and (c) accumulation of sperm specific histone H3.10 which is resistant to K27 methylation in *Arabidopsis* (Borg et al., 2020; Vigneau and Borg, 2021). Removal of H3K27me3 during spermatogenesis leads to the transcription of embryogenesis-related genes, namely, LEAFY COTYLEDON 1 (LEC1) and BABY BOOM (BBM). BBM was originally identified as an AP2 domain transcription factor in the differentiated somatic cells responsible for modulating embryogenesis (Boutilier et al., 2002). Therefore, paternal H3K27me3 reprogramming is considered to prepare for sporophytic phase transition and may influence transcription in early embryogenesis. In *B. napus*, H3 and H4 acetylation levels were prevalent in the early stages of microspore development but as the pollen matures, repression of *BnHAC5-like* gene causes decreases in histone acetylation levels (Pérez-Pérez et al., 2025). Histone acetylation and DNA hypomethylation play a key role in microspore embryogenesis by enhancing chromatin decondensation and providing easy access to the transcription factors of developmental organization (Ahmadi et al., 2018).

### Embryogenesis

Plant embryogenesis is driven by the asymmetric apical-basal axis development in the zygote as denoted in *Arabidopsis*. YODA (paternally activated MAPKK Kinase) and WOX8 (WUSCHEL-related homeobox 8) coordinate asymmetric zygote division through the YDA-WRKY2-WOX8 signaling cascade (Frost et al., 2024). While this signaling cascade is well established in *Arabidopsis*, its orthologs are yet to be identified in *Brassica*. Auxin production involves a two-step pathway - (i) through TAA1 (TRYPTOPHANE-AMINOTRANSFERASE OF ARABIDOPSIS 1) and related TAR1/TAR2 enzymes, and (ii) YUCCA monooxygenases. YUCCA transcription is methylation-dependent and may be mediated by several classes of methyltransferases. YUCCA6 was reported to modulate branch angle in *B. napus* but chromatin modification by methyltransferases is yet to be explored. Alternatively, the chromatin remodeling factor, *BrCHR39*/*Bra014815* homolog of *AtCHR39*, is known to positively regulate apical dominance by auxin signaling by targeting *BrASK18* (Arabidopsis Skp-1 like 18) in *Brassica rapa* (Liu et al., 2025).

Loss of CHH methylation through the RdDM pathway led to seed deformity in *B. rapa* (Chakraborty et al., 2021). In *B. rapa*, embryo hypermethylation was achieved through the RdDM pathway at both euchromatic and heterochromatic positions. Chakraborty et al. demonstrated that the autonomously derived siRNAs drive the hypermethylation in the embryos. Successful development of seeds in the *rdr2* mutant despite absence of embryo hypermethylation highlighted that maintenance of methylation may not be necessary for embryogenesis (Chakraborty et al., 2021). siRNAs produced in the maternal somatic tissue might migrate to the embryo or the endosperm to target transposon-associated genes like AGAMOUS-like transcription factors and modulate seed development (Grover et al., 2020). In *Arabidopsis*, Endosperm maintains hypomethylated state of the maternal alleles by the demethylation activity of DME (Kordyum and Mosyakin, 2020). Such uniparental-specific gene expression patterns, known as genomic imprinting, have been identified during endosperm development (Huh et al., 2007). It has been proven to be influenced through asymmetric DNA methylation and histone repression (Liu et al., 2018; Yoshida et al., 2018). However, embryo hypermethylation did not correlate with the hypomethylated state of the endosperm in *B. rapa* (Chakraborty et al., 2021).

In *Brassica napus* BnHAC5 (CBP family) plays a pivotal role as a positive regulator of stress-induced microspore reprogramming and may activate embryogenesis genes essential for somatic embryogenesis (Pérez-Pérez et al., 2025). Through the Enhanced levels of H3K9me2, H3Ac and H4Ac were involved in transcriptional activation and proliferation during microspore embryogenesis in *B. napus*, suggesting the role of *Bn*HKMT and *Bn*HAT in somatic embryogenesis (Rodríguez-Sanz et al., 2014). Blocking the function of BnHDACs also induced haploid embryogenesis suggesting the importance of HDAC-dependent mechanisms and hyperacetylation of H3 and H4 histones in microspore embryogenesis (Li et al., 2014).

### Polyploid evolution

Polyploidization drives adaptation and domestication by reshaping the transcriptome, proteome and metabolome landscape in angiosperms (Barker et al., 2024). Depending on the origin and composition of the chromosome, whole-genome duplication produces autopolyploids (single genome doubling) or allopolyploids (merging of diverging genomes) (Chen, 2007). Gene duplication in autopolyploids primarily disturbs dosage regulation, whereas in allopolyploids, interspecific hybridization triggers both genetic and epigenetic changes. Such reversible epigenetic regulations including DNA methylation and histone modification, influence the genomic balance, hybrid compatibility, and facilitate diploidization. Dominant genomes contribute more to the evolution and homeolog expression patterns in a phenomenon called subgenome dominance (Li et al., 2020a; Wang et al., 2022). Accumulation of new mutations in one of the multiple gene copies gives an added advantage by liberating genes from selection in many polyploids. *Brassica*’s lineage (U triangle) substantiates this process where *B. napus*, *B. carinata*, and *B. juncea* evolved from the interspecific hybridization and chromosomal doubling of the diploid progenitors - *B. rapa*, *B. oleracea* and *B. nigra* (Wang et al., 2025b).

Differential cytosine methylation patterns often emerge in polyploids causing reversible modifications in the gene expression pattern, transposon control, and subsequent genome stability. Allopolyploidy causes re-patterning in cytosine methylation over generations. *B. oleracea*, a diploid species, ancestor to *B. napus*, is more similar in its patterns of these modifications to the allotetraploid species over other diploid ancestral species, such as *B. rapa* (Liu et al., 2014b). These dissimilarities in DNA patterns and histone methylation can be due to genomic structure and localization of heterochromatin instead of ploidy level in *Brassica* species (Braszewska-Zalewska et al., 2010). Certain subsets of orthologous genes may get silenced through these epigenetic modulations. Nuclear dominance through unequal contribution of the parental genome is caused by the cytosine methylation mediated rRNA loci repression (Tucker et al., 2010). siRNA accumulation and cytosine methylation were ubiquitously distributed in the C subgenome leading to its repressed contribution than A subgenome in *B. napus* seeds (Ziegler et al., 2023). Epigenetic changes in the CpG methylation is upregulated in *B. napus* (AACC) in comparison to its diploid progenitors and remains fixed throughout generation T0 to T5, persisting over generations to affect C genome. Such changes are tightly regulated to support heterochromatin formation. While the loss of CMT3 is linked to the loss of the gbM genes in other species, however, reduced levels of mCG within gbM did not correlate with the presence of CMT3 in certain *Brassica* species (*B. oleracea*, *B. rapa*, and *S. parvula*). One of the reasons this can be attributed to is the evolution of null alleles disrupting CMT3 function, thus, leading to fewer gbM genes and methylation levels across the *Brassica* family. DNA methylation was also reported to be present exclusively in the heterochromatin region of *B. rapa*, while in *B. oleracea* and *B. napus* it was detected across both euchromatin and heterochromatin regions (Bewick et al., 2017).

Nuclear dominance is also promoted synergistically through repressive histone modifications at the uniparental rRNA loci (Chen and Pikaard, (1997a)Lawrence et al., 2004). *B. oleracea* rRNA genes are repressed and rRNA genes of *B. rapa* remain active in *B. napus*. Silenced loci hold potential to activate in floral organs without meiotic resetting, indicating the role of developmental plasticity in overriding epigenetic silencing (Chen and Pikaard, (1997b)). The silenced state is catalyzed by the nucleolar histone deacetylase HDT1 which causes H3K9 deacetylation and subsequent methylation. Transcriptome-wide m^6^A methylomes of *B. napus* and its diploid progenitors, *B. rapa* and *B. oleracea* revealed distinct distribution of the four epigenetic markers - H3K4me3, H3K27ac, H3K27me3, and DNA methylation, contributing to the functional divergence of the tandem and proximal duplicates (Li et al., 2023b). Therefore, these epigenetic modifications cause nuclear enlargement, genomic rearrangement, and transcriptional reprogramming to influence phenotypic diversity in polyploids.

### Stress resilience

Epigenetic modification provides rapid response to various biotic and abiotic stressors and may even provide quicker future adaptations than genetic mutations. These stress-induced heritable modifications hold potential to be retained within-generation and by the next generation as ‘stress memory’ for subsequent stress adaptations in climate-smart cultivars.

### Abiotic stress

The MSAP (Methylation-Sensitive Amplified Polymorphism) approach was used to identify salt and heat induced DNA methylation variation in the tolerant and sensitive rapeseed cultivars (Marconi et al., 2013; Gao et al., 2014; Guangyuan et al., 2007). Methylome and transcriptome profiling revealed heat-induced hypermethylation in the promoter region of ribosome biogenesis related genes causing abnormal flowering in *B. oleracea* (Yao et al., 2022). Functional roles of DNA demethylase were also identified in heat and salt stress in rapeseed. Expression profiles of DMEa and DMEb were uniformly low across major tissues, except in the ovules 15 days after flowering. Alternatively, ROS1a and ROS1b expression increased in every tissue except the mature seeds. Barring ROS1a, all the remaining demethylases denote increased expression under heat and salt stresses indicating abiotic stress can induce DNA methylation variation in *B. napus* (Fan et al., 2020). Genome-wide identification of 92 m^6^A-regulatory genes in *B. napus* highlighted functional divergence through tissue-specific expression patterns in response to external factors (Shan et al., 2025). Role of m^6^A modification in heat stress resilience of *B. rapa* was detected using MERIP-Seq with the 3’ UTR enriched with m^6^A peaks in the contrasting cultivars (Liu et al., 2020). The study further established a relationship between m^6^A RNA methylation and gene expression levels confirming m^6^A role in conferring stress tolerance. Similar study linking m^6^A mRNA methylation with cold stress tolerance revealed distribution of higher 5’UTR m^6^A in cold-resistant variety and conferred tolerance by regulation of gene expression patterns of cold responsive genes. Analysis of the gene expression profile and m^6^A methylation peak of zinc finger protein ZAT12 (Zinc Finger of *Arabidopsis Thaliana* 12) indicated positive regulation to improve cold tolerance (Ma et al., 2025). Differentially regulated siRNAs modulate heat tolerance in B. campestris by targeting several temperature and developmental processes-associated genes (Ahmed et al., 2021).

In *Brassica rapa*, HATs and HDACs not only maintain the histone acetylation-deacetylation balance but also regulate seed germination and peroxidase activity under low temperature to improve stress resistance (Bian et al., 2024). Reactive oxygen species (ROS) triggered S-nitrosylation of HDACs may establish stress memory and improve tolerance for future stress conditions (Kaya and Adamakis, 2025).

HD2s can also associate with HD2C to modulate stomatal closure and root growth under drought stress through ABA and salt stress responsive pathways (Luo et al., 2012). Epigenetic modifications influence several phytohormone signaling pathways to adapt to external stressors (Kaya et al., 2024). In *B. rapa*, *B. napus* and *B. oleracea*, 8, 14, and 10 HDT genes displayed organ-specific expression (flowers, buds and siliques) at different developmental stages under ABA and ethylene treatment, however their functional validation is pending (Xie et al., 2022). Evolutionary and expression studies reveal the role of BraHDA3 in heat stress, a homolog of AtHDA14 responsible for upregulating the proline level (Eom and Hyun, 2021). Chromatin accessibility enabled salt stress-responsive genes to exert heterotic effects by combining the advantageous alleles (Chen et al., 2025b). Thermal Resistance Gene 1 (*Bn*TR1), an E3 ubiquitin ligase, enhanced thermotolerance in *B. napus*, classifying it as an important candidate for marker-assisted breeding (Liu et al., 2014c).

Overexpression of Bna-miR399c prompted down-regulation of BnPHO2 and up-regulation of key phosphate transport and signaling genes (BnPHR1, BnPHF1 and PHT1 family). This led to significant increase in the taproot length and lateral root number with a spike in Pi accumulation under low Pi stress but (Fujii et al., 2005; Chiou et al, 2006; Lin et al, 2008). Accordingly, Bna-miR399c improves transportation and absorption of Pi in soil, enhancing low Pi stress resistance in *B. napus* (Du et al, 2023). This validates that Brassicaceae and its miR399-PHO2 module are functionally conserved and can be used for future generation of sustainable stress-resilient mustard through genome editing and breeding programmes as it effectively regulates the homeostasis of Pi in *B. napus* and opens a pathway for germplasm innovation and the creation of cognitive crops with low nutrient input and high yield (Du et al., 2023; Wang et al., 2004b; Ayadi et al., 2015).

### Biotic stress

Recently, the regulatory role of DNA methylation in response to white rust was indicated in *B. rapa* through whole-genome DNA methylation analysis. 233 and 275 differentially methylated regions (DMRs) were identified in which the CG sites showed maximum methyl group enrichment (Tirnaz et al., 2022). Differential methylated patterns were also observed in the promoters of defence genes involved in blackleg disease of *B. napus*, indicating the regulatory role of DNA methylation in conferring biotic stress tolerance (Tirnaz et al., 2020). Foliar herbivory in *B.rapa* induced DNA demethylation and phenotypic changes including morphology, flower number and scent, which notably reduced its attractiveness to pollinators. In *Brassica rapa*, BrHDA6 functions to enhance non-histone deacetylation of Sulphotransferase 12 (BrSOT12) to induce downey mildew resistance (Wang et al., 2025c). Additionally, differentially expressed miR5139, miR159, and miR390 led to cleavage of several genes including disease resistance associated genes in response to *S. sclerotiorum* infection in *B. napus* (Regmi et al., 2021). *Brassica* miRNA, miR1885, regulates both plant growth and innate immunity by silencing TIR-NBS-LRR-type resistance gene (BraTNL1) and represses the expression of CHLOROPHYLL PROTEIN 24 (BraCP24) through trans-acting silencing (Cui et al., 2020). Thus, making it an ideal target for future breeding strategies.

Important regulators involved in the maintenance of the epigenetic machinery in the *Brassica* family have been detailed in **Table 1**.

**Table 1:** Components of epigenetic mutation in the *Brassica* family.

S no	Epigenetic modification	Gene/protein	Function	Trait	Species	Reference
1	DNA methylation	MET1	DNA methyltransferase	male germline development	*Brassica rapa*	Zhang et al., 2025
2		CMT2	DNA methyltransferase			
3		ROS1	DNA demethylation			
4		CMT3	DNA methyltransferase	Evolution of gene-body methylation	*Brassicaceae*	Bewick et al., 2017, Plskova et al., 2024
5		DMR	DNA methylation	Biotic stress response (white rust disease)	*Brassica rapa*	Tirnaz et al., 2022
6		DMR	DNA methylation	Biotic stress response (blackleg disease)	*Brassica napus*	Tirnaz et al., 2020
7		RDR2	RNA-dependent RNA polymerase 2	Embryogenesis	*Brassica rapa*	Chakraborty et al., 2021
8		DMEa & b	Demeter (DNA demethylation)	Heat & salt stress response	*Brassica napus*	Fan et al., 2020
9		ROS1a & b	Repressor of Silencing 1			
10		m6A-regulatory genes	RNA methylation	Polyploid evolution	*Brassica napus, Brassica rapa, & Brassica oleracea*	Li et al., 2023
11				Polyploid evolution	*Brassica napus*	Shan et al., 2025
12				Heat stress response	*Brassica rapa*	Liu et al., 2020
13				cold stress response	*Brassica rapa*	Ma et al., 2025
14	Histone acetylation	HAC1	Histone acetyltransferase	Regulate flowering	*Brassica rapa*	Si et al., 2021
15		HAC5	Histone acetyltransferase	Microsopore reprogramming	*Brassica napus*	Pérez-Pérez et al., 2025
16	Histone deacetylation	HDT1(HD2A)	Histone deacetylase	Hormonal and Stress response	*Brassica rapa, Brassica napus and Brassica oleracea*	Xie et al., 2022
17		HDT2(HD2B)				
18		HDT3(HD2C)				
19		HDT4(HD2D)				
20		HDA3		Heat stress response	*Brassica rapa*	Eom and Hyun, 2021
21		HDA6		Downey mildew tolerance via non-histone mechanism		Wang et al., 2025c
22		SRT1		Putative early flowering		Eom and Hyun, 2021
23		SRT2		Putative early flowering		
24	Histone methylation	H3K27me3	Histone methyltransferase	Regulate flowering under cold stress response	*Brassica rapa*	Akter et al., 2019; Payá-Milans et al., 2019
25		H3K34me3		Regulate flowering		Mehraj et al., 2021a
26		H3K36me3				
		H3K4me3		Modulates the expression of BnFTA2 to regulate flowering time	*Brassica Napus*	Abelenda et al., 2023; Del Olmo et al., 2019
		CLF (PRC2 component)		Regulates flowering by repressing FLC gene expression	*Brassica rapa*	Shen et al., 2021
		SDG8		Regulates flowering by repressing FLC gene expression	*Brassica rapa, Brassica Napus*	Fu et al., 2020; Jiang et al., 2018
27	Histone demethylation	Jumonji H3K27me3 -REF6, ELF6	Histone demethylase	Regulates flowering	*Brassica rapa*	Poza-Viejo et al., 2022
28	Chromatin remodeling complexes	BrCHR39/Bra014815 (AtCHR9 homolog)	SHPRH subfamily	Apical dominance	*Brassica rapa*	Liu et al., 2025
29	RNA-Directed Gene Silencing	Bna-miR399c	microRNA	Improved Pi uptake, low Pi stress tolerance	*Brassica napus*	Fujii et al., 2005; Chiou et al., 2006; Lin et al., 2008; Du et al., 2023
		DE-siRNA	siRNA	Heat tolerance	*B. campestris*	Ahmed et al., 2021
		miR5139, miR159, and miR390	microRNA	*S. sclerotiorum* infection (Biotic stress response)	*B. napus*	Regmi et al., 2021
		miR1885	microRNA	Modulates pathogen response and growth development	*B. rapa*	Cui et al., 2020
		BrFLC2as	Long non-coding RNA (lncRNA)	Represses FLC expression (improves flowering time)	*Brassica rapa*	Hepworth and Dean, 2015

## Current advances in epigenetic technologies for modulating developmental regulations

### Application of high-throughput sequencing

The introduction of high-throughput sequencing (HTS) platforms has revolutionized epigenomic research by allowing for unbiased, magnified, comprehensive, and high-resolution mapping of DNA methylation patterns across whole genomes. These approaches have improved the capacity to associate methylation to transcriptional regulation, chromatin remodeling, and trait expression in crops (Talarico et al., 2024; Kumar and Mohapatra, 2021; Kumari et al., 2022). In monocots, methylation variants correlate with yield heterosis and stress adaptability, suggesting that epialleles may contribute to trait diversity beyond genetic polymorphisms in diploids as well (Talarico et al., 2024; Kakoulidou et al., 2021; Sun et al., 2018). Bisulfite sequencing (BS-Seq) is the “gold standard” approach as it provides simple and efficient detection of methylation patterns with single-base precision, as demonstrated by several studies (Cokus et al., 2008; Feng et al., 2010; Gent et al., 2013; Li et al., 2020b). The global DNA methylation patterns of the rapeseed genic male sterile line 7365A and its near-isogenic fertile line 7365B were compared using Whole genome bi-sulphite sequencing (WGBS). Genome-wide DNA methylation profiling denoted lower methylation levels in floral buds than leaves and roots (Wang et al., 2018). Tirnaz et al., 2022, performed WGBS and differentially Methylated Regions Analysis (DMRs) to analyze possible regulatory function of DNA methylation modification in defensive mechanisms that could be used to enhance biotic stress resistance (Tirnaz et al., 2022).

Reduced representation bisulfite sequencing (RRBS) uses methylation-insensitive restriction enzymes and size selection to generate genome fractions for bisulfite conversion and next-generation sequencing (NGS). Low-cytosine-coverage RRBS in plants has been used to explore methylation in *B. rapa* sub-genomes and Quercus promoters under temperature regimes. Chen et al. (2015) employed a modified RRBS approach to generate a genome-wide DNA methylation profile of *B. rapa*. These findings shed new perspective on the role of epigenetic variation in the evolution of polyploid genomes and suggest a novel mechanism for duplicate gene removal. Modified RRBS sampling profiled 2.24% of CG, 2.16% CHG, and 1.68% CHH sites in *B. rapa* affirming hierarchical methylation and transcription across sub-genomes (Mehraj et al., 2021b). Besides, RRBS discovered conserved and divergent methylation patterns associated with environmental adaptation and plant methylation evolution (Hsu et al., 2017; Nkongolo and Michael, 2024). Genome-wide profiles of DNA methylation and histone modification landscapes in *B. napus* were analyzed under salt stress and then merged with RNA-seq (Kong et al., 2025). These findings denoted that H3K27me functions antagonistically to H3K4me and suppressed gene expression of several salt stress-associated genes, implying that epigenetic modulations in *B. napus* are key for modulating stress response.

Study of DNA methylation regions promotes identification of methylation polymorphism which are capable of influencing phenotypic traits. Methylome sequencing revealed that heat stress induced DNA hypomethylation in the microspores of *B. napus*. Heat stress treatment enhanced CHG differential DNA methylation owing to overlap between the transposable elements and hypomethylated regions (Li et al., 2016). Among conventional methylation methods, MSAP (using HpaII and MspI) remains widely applied across several species including *B. napus* (Li et al., 2015; Xu et al., 2015; Gimenez et al., 2016a, b; Wang et al., 2016a; Kumar et al., 2016; Li et al., 2017; Abid et al., 2017). Salmon et al. evaluated 30 *B. oleracea* populations using MSAP to relate DNA methylation with phenotypic variability (Salmon et al., 2008). However, MSAP lacks sequence-context resolution. To overcome limitations in complex genomes, Methylation Sensitive Amplification Polymorphism Sequencing (MSAP-Seq) provides the simultaneous identification of hundreds of thousands of sites at a minimal cost.

HTS-based small RNA-seq and long-read transcriptomics have expanded understanding of regulatory RNAs. In *Brassica species*, small RNAs regulate glucosinolate biosynthesis, seed-oil accumulation, and photoperiod-dependent flowering. Natural epialleles modulated by RNA-mediated methylation have been identified in *B. rapa* and linked to stress recovery, pathogen defense, and nutritional composition (Chow and Mosher, 2023). Prediction of complex traits requires integration of genomic, transcriptomic, and methylomic data. *B. rapa* multi-epigenetic analyses (siRNAs, DNA methylation, H3K27me3, lncRNAs) identified candidate lncRNAs under epigenetic regulation. Transcriptome and histone-modification changes in dehydrated rapeseed showed genome-scale alterations in expression and H3K4me3/H3K27me3 patterns, especially in P5CS (Delta-1-pyrroline-5-carboxylate synthetase) genes. Targeted bisulfite sequencing revealed stress-dependent gene-body methylation in BnP5CSA.

ChIP-seq has mapped dynamic histone landscapes associated with activation (H3K4me3, H3K9ac) or repression (H3K27me3) (Kong et al., 2025). Histone marks on flowering genes such as FLC, FT, and SOC1 influence phenology and resource allocation. H3K27me3 ChIP-seq experiments were performed to identify the role of BraA.CLF as a histone methyltransferase in *B. rapa* (Poza-Viejo et al., 2024). Assay for Transposase-Accessible Chromatin (ATAC)-seq in *Brassica* is emerging with regulatory modules associated with lipid metabolism, sulfur absorption, secondary metabolite biosynthesis, and reproductive transition. ATAC-Seq identified allopolyploidization induced chromatin accessibility in *B. napus*. Accessible chromatin regions (ACRs) associated with H3K27me3 were relatively more accessible than the ones with H3K4me3 or H3K27ac modifications (Li et al., 2022b). H3K27me3 ChIP-seq and 3’ RNA-seq further mapped developmental chromatin dynamics in B. rapa leaves and inflorescences.

Single-cell genomics and epigenomics have advanced substantially, enabling profiling of cell-specific variants and epigenetic features in plant models. Bulk chromatin accessibility has been mapped in *Arabidopsis*, rice, and maize, though single-cell chromatin studies remain limited. Trans-factor binding discovery relies heavily on ChIP-seq requiring many input cells, while epigenetic and epitranscriptome modifications shape developmental programs, environmental responses, and evolutionary adaptation (Luo et al., 2020). Adapted Hi-C technologies (scHi-C) allow single-cell resolution of chromatin architecture. These technologies can be applied in *Brassica* where subgenome dominance complicates regulatory pathways. Single cell approaches will reveal spatial-temporal activity of epigenetic modulators and may shed light on homeolog functionality and hybrid variability.

### Epibreeding in *Brassica*

Epibreeding utilizes heritable epigenetic variation such as DNA methylation, histone modifications, and small RNA-guided chromatin states for sustainable crop improvement (Dalakouras and Vlachostergios, 2021). In *B. napus*, *B. rapa* and *B. oleracea*, genome-wide methylome surveys reveal the presence of abundant natural epigenetic diversity due to whole-genome triplication and allopolyploid evolution (Feng et al., 2022; Li et al., 2022b; Hu et al., 2023). Epigenomic changes during polyploidization and hybridization, especially in CG/CHG methylation and small RNA levels, drive subgenome dominance and gene expression alteration (Chen et al., 2015). These phenomena directly influence phenotype and adaptation (Li et al., 2019). Important agronomic traits such as flowering time, seed oil composition, and stress tolerance, are affected by stable epialleles or methylation-dependent regulation (Gautam et al., 2016; Li et al., 2023; Zheng et al., 2022; Poza-Viejo et al., 2024; Hu et al., 2023). Epibreeding in *Brassica* involves epigenetic diversity mining, where methylation-sensitive markers, MSAP profiling, and WGBS helps in identifying stress-responsive or developmentally regulated epialleles showing transgenerational stability (Gautam et al., 2016; Wang et al., 2016b). Canola epilines were generated through consistent selection over three generations for enhanced energy use efficiency and drought tolerance (Verkest et al., 2015). The selected epilines denoted elevated expression of H3K4me3 associated stress responsive genes. These markers can be used in epi-QTL mapping to enable selection based on chromatin activity rather than DNA sequence alone (Springer and Schmitz, 2017). Such as in *Brassica napus*, 125 epiQTLs associated with seven agronomic traits (seed yield, oil content, glucosinolate content, flowering time, plant height, and branch number) have been identified (Long et al., 2007; Shi et al., 2009; Long et al., 2011). These epiQTLs showed methylation-based variation in centromeric and transposon regions (Long et al., 2011). Similarly, seven robust QTLs associated with pod shatter resistance on A02, A03, A05, A09 and C01 have been identified, which are located near key regulatory genes such as FUL (*FRUITFULL)* (Raman et al., 2023). With most epialleles showing 90–97% genetic stability across environments and generations, epiQTL represent reliable heritable targets for selection (Long et al., 2011). Utilization of these epiQTLs for epibreeding based *brassica* improvement programs have been an unexplored area and therefore it provides a conceptual framework to enhance resilience and productivity while conserving genome integrity. Laying the foundation for sustainable improvement of *Brassica* species using the epigenetic factors.

### Role of epigenome editing in *Brassica*

**Figure 3:**
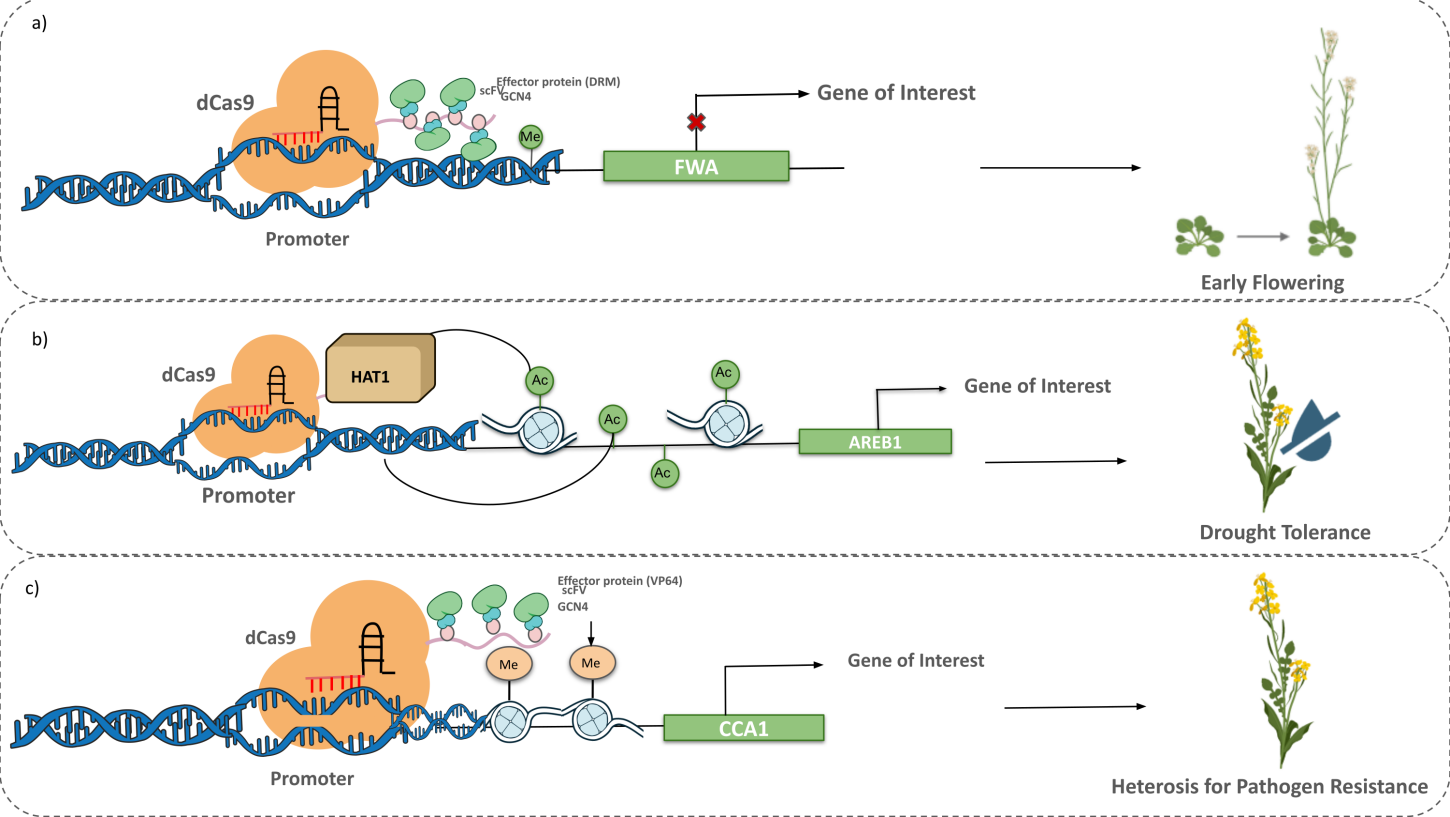
Role of epigenome editing in *Brassica*. **(a)** CRISPR-dcas9/SUNTAG system with DRM methyltransferase as the effector protein can be used to repress FLOWERING WAGENINGEN (FWA) gene following methylation in its promoter region to induce early flowering. **(b)** dCas9 fused with Histone Acetyltransferase (dCas9-HAT) system can be used to target promoter region of abscisic acid (ABA)-responsive element binding protein 1 (AREB1) to drive its overexpression for enhanced drought tolerance. **(c)** Transcriptional activation using the CRISPR-dcas9/SUNTAG system fused with histone methyltransferase SET DOMAIN GROUP 2 (SDG2) of CIRCADIAN CLOCK ASSOCIATED 1 (CCA1) gene can be achieved by inducing H3K4me3 in its promoter region.

Epigenetic sequencing distinguished the key epigenetic alterations correlated with the desirable traits paving the way for epigenomic-assisted selection or “epi-breeding”. However, this mode of selection relies on non-specific natural or induced variations which can be overcome through targeted genome editing by using site-specific nucleases, namely, Zinc-Finger Nucleases (ZFNs), Transcription-Activated Like Effector Nucleases (TALENs), and Clustered Regularly Interspaced Short Palindromic Repeats (CRISPR) based systems. Several studies reviewed the role of epigenome editing and its application in plants (Leech et al., 2025; Qi et al., 2023; Sen et al., 2025). Precise alteration through next-generation CRISPR/Cas systems hinges on a (i) nucleotide binding guide RNA (gRNA) which directs the (ii) Cas protein to induce double-stranded break at the target location. Recently, the technology has been repurposed to generate fusion proteins with modified epigenetic enzymes or their catalytic domain as exemplified in **Figure 3**. Catalytically inactive or ‘dead’ Cas9 could repress gene expression by hindering the binding of the RNA Polymerase activity, which is now referred to as CRISPR interference (CRISPRi). The CRISPR/dCas9 system integrates epigenetic effectors including DNA methyltransferase, demethylase, or histone modifiers with dCas9 protein to create epigenetic alterations at the endogenous locus without introducing permanent genetic mutations (Molla et al., 2021). For instance, the Krüppel-associated box (KRAB) domain of Kox1 is a strong transcription repressor, and is implemented for gene repression. Contrarily, CRISPR activation (CRISPRa) utilizes effector domains, including Herpes simplex viral protein (VP64), VP16, or transactivator domain of nuclear factor kappa B (p65).

In *Arabidopsis*, researchers have successfully employed CRISPR/dCas9 systems fused with epigenetic modifiers like DNA methyltransferases (DNMTs) and demethylases (Ten Eleven Translocations 1, TET1) to add or remove chemical modifications and control gene expression in a targeted and heritable manner (Gallego-Bartolomé et al, 2018). An adapted epigenome editing technique with enhanced on target methylation efficiency, CRISPR-cas9 SunTag systems, involves fusion of dCas9 with a chain of General Control Nonderepressible 4(GCN4) peptide epitopes, where each repeat is connected to a transcription regulator through an anti-GCN4 antibody known as Single Chain Fragment Variable (scFv). SunTag is based on the principle of enhanced transcription activation due to combination of multiple transcription factors with a single promoter. Building on this foundational work in *Arabidopsis*, epigenome editing is being applied to *Brassica* crops, which are closely related to *Arabidopsis* and share a high degree of genome synteny. Controlling yield- related characteristics like flowering time, seed oil content, improving hybrid yield, and glucosinolate production is another important application area. For instance, epigenetic approach showed a reliable method to control the expression of genes encompassed in stress resistance which includes salinity, drought, heavy metal, cold, submergence tolerance (Benoit et al., 2019). These alterations by activating or silencing critical defense genes create specific indicators that could be beneficial for the next generation (Dai et al., 2015). Alternations in the DREB1A or RD29A via epigenetic changes showed enhancement to drought stress in rice genotypes. To induce early flowering in *brassica* epigenetic modulation such as silencing of FWA (FLOWERING WAGENINGEN) gene, which is responsible for delay in flowering time could be implemented like in *Arabidopsis* (Soppe et al., 2000). A framework for modifying important agronomic traits in *Brassica* without changing DNA sequences is provided by methods shown in *Arabidopsis*, such as dCas9-SUNTAG system mediated DNA methylation or dCas9-KRAB induced repression. However, challenges such as delivery into polyploid genomes, off-target epigenetic effects, and ensuring heritability of desired epigenetic marks must still be addressed for widespread agricultural application.

## Conclusion and future perspectives

As sessile organisms, plants face an unprecedented uphill task to defend themselves from the environmental stressors while maintaining yield and nutritional quality. By understanding the underlying epigenetic mechanisms to pin genomic loci that undergo transcription modulation in response to environmental factors will pave the way for developing improved *Brassica* cultivars. Heritable epigenetic changes leading to activating the previously transient molecular switch will be beneficial in modulating the plant’s stress responses. Establishment of DNA methylation and histone modification patterns has been identified to influence polyploidy genome architecture and stress tolerance patterns in the *Brassica* family. Notably, comparative histone modification studies reveal clear knowledge gaps: although histone phosphorylation has been studied in *Arabidopsis*, it has not yet been identified in rapeseed mustard and related *Brassica* species. Histone ubiquitination remains an unexplored domain whereas histone sumoylation is a completely new domain to explore in both *Arabidopsis* and other *Brassica* species. Mechanism of H3K79 methylation is well defined in mammals, but its existence in plants is yet to be established. Future work could entail identification of orthologs in plants, specifically *Brassica*, to identify if these marks influence hybrid variability. Moreover, the repertoire of HATs and HDACs appears less diverse in rapeseed -mustard compared to *Arabidopsis*, suggesting possible lineage-specific reductions in chromatin regulatory machinery. These gaps highlight an urgent need for systematic functional characterization of histone modifiers in the polyploid *Brassica* genome. Several factors have been identified to modulate regulatory pathways, for instance, RVE8/LCL5 controls acetylation of TOC1. While their orthologs exist in *Brassica*, the underlying epigenetic mechanisms are yet to be confirmed if conserved. SRT1 modulates flowering by acetylation, but its role in ethylene regulation has also been recently highlighted. It will be interesting to understand where these pathways converge and if they are epigenetically modulated. Similarly, it will be noteworthy to identify epigenetic impact on the polyploid evolution of *Brassicas* and the role it plays in conferring stress tolerance. Despite the advancement in multi-omics techniques for discovering novel target sites, establishment of comprehensive regulatory networks is still pending in *Brassica*. Analyzing epigenome marks combined with recent single-cell technologies like INTACT may provide a complete picture of the fundamental regulatory pathways, particularly during gametogenesis and embryogenesis. Utilization of recent advancements such as APOBEC-coupled epigenetic sequencing and the CRISPR/dCas9 based epigenome editing will propel crop improvement and provide the basis for marker-assisted breeding. Additionally, crosstalk between the nuclear and mitochondrial methylation will prove advantageous for providing multi-level epigenetic adaptations.
